# Investigation of genetic variation in *Jatropha curcas* by Ecotilling and ISSR

**DOI:** 10.1186/1753-6561-5-S7-O50

**Published:** 2011-09-13

**Authors:** Fatemeh Maghuly, Joanna Jankowicz-Cieslak, Alberto Calari, Rose Ramkat, Bradley Till, Margit Laimer

**Affiliations:** 1 Plant Biotechnology Unit (PBU), Dept. Biotechnology, University of Natural Resources and Life Sciences, Muthgasse 18, 1190 Vienna, Austria; 2 Plant Breeding Unit, FAO/IAEA Agricultural and Biotechnology Laboratory, International Atomic Energy Agency, P.O. Box 100, Vienna International Centre, 1400 Vienna, Austria

## Background

The ability of species to adapt to different environments resides in their genetic diversity. This diversity, most commonly manifested as Single Nucleotide Polymorphisms (SNPs), can provide clues to the adaptive processes and population histories that have played a role in the species’ evolution. A number of different techniques for identifying SNPs have been developed, all having their limitations.

Reverse genetics approaches rely on the detection of sequence alterations in target genes to identify allelic variations in natural or mutant populations. Ecotilling, a variant of TILLING (Targeting Induced Local Lesions IN Genomes) technique, allows high-throughput analyses of natural genetic diversity in plants [1], particularly in species with limited genetic diversity.

*Jatropha curcas* L. is a perennial, monoecious shrub of the *Euphorbiaceae* family, native to America but distributed widely in the tropical and subtropical areas [2]. Wild or semi-cultivated types of *J. curcas* can grow well under unfavourable climatic and soil conditions [3]. *J. curcas* has attracted a great deal of attention worldwide, regarding its potential as a new energy plant. The seeds of *J. curcas* contain 30-45% oil [4] with a high percentage of monounsaturated oleic and polyunsaturated linoleic acid [5]. For genomic analyses, *J. curcas* is an interesting model species, since it has a relatively small genome (2C DNA content of 0.850 ± 0.006 pg or C DNA content of 0.416 × 109 bp) [6].

However, to achieve specific breeding goals in *Jatropha* for wider ecological adaptation, disease resistance and novel seed quality, the use of germplasm from different group and regions is necessary. Understanding the population structure of the alternative bioenergy plant *Jatropha curcas* is challenging due to limited genetic variability and information on phylogenetic relationships between accessions and related species. The development of cultivars of *Jatropha curcas* by conventional breeding will profit largely from biotechnological support (pathogen-free accessions with specific traits, non-toxic, high yielding varieties).

The knowledge about *J. curcas* remains limited and little genomic research has been done so far [7]. In fact, the genetic map of *J. curcas* is not well-developed and only few molecular markers exist that could be used to clearly distinguish world wide accessions. Therefore, a resource database of SNPs in *J. curcas* would provide researchers with a tool for answering questions concerning population structure or adaptation and allow comparison of this species with related species.

## Methods

The identification of novel SNPs that account for natural variation was used to study genetic diversity and the relationships between and within *Jatropha* species. ISSRs (*Inter* Simple *Sequence* Repeats) also were considered as a tool in selecting germplasm for breeding purposes.

An *in vitro* germplasm collection of 1300 accessions from 12 countries was established. This collection will serve different purposes: a) conserve valuable genetic resources, b) survey genetic variation, and c) serve as starting material for genetic improvement with different breeding goals.

Ecotilling was applied to 12 different genes of interest related to stress tolerance, toxin and oil metabolism. 50 ISSR primers were used to assess the genetic diversity of *Jatropha curcas* and related species. Four different pooling strategies were used to identify homozygous and heterozygous SNP variations. In fact, variation was analyzed both within a single tree (heterozygous) as well as between individual trees and a reference samples. Due to the reported low variations between *Jatropha* accessions [8,9] and large size of our collection, the 8 x 8 pooling strategy was chosen to estimate the level of variations among 12 selected genes.

## Results and conclusions

To elucidate genetic relationship among *Jatropha* accessions from different regions and related species, a dendrogram was produced using NJ analysis of NeiÂ´s genetic distance for 5 ISSR markers. The dendrogram is divided into two groups, one containing all *Jatropha* accessions and the other containing the related species. The main *Jatropha curcas* cluster is divided into two subclusters, one containing samples from Kenya and the other containing the remaining *Jatropha* accessions. The data showed clear variations not only among individuals but also between different regions.

Ecotilling was found to be more efficient for large-scale studies of genetic variation in *Jatropha*, compared to RAPD, SSR and AFLP. Ecotilling is a low cost, high-throughput reverse genetic method for haplotyping and SNPs discovery.The level of differentiation observed was based on the geographic distribution pattern, i.e. it was higher in the centre of origin. ISSR analysis yielded highly reproducible patterns with 5/50 primers.
